# Mind and Health

**Published:** 1879-03

**Authors:** 


### MIND AND HEALTH.

Women, the world over, are inquisitive, very; as largely so out of their own sphere as in it. A lady correspondent desires to know what the diary of Mr. and Mrs. Sparrowgrass, in a previous number, had to do with health? If courtesy had not forbidden, I might have referred her to a Down Easter, on his way to Boston, who is said to have been questioned by an acquaintance thus:

*Friend.*—Why Eb! where are you going to-day?

*Ebenezer.*—To Boston.

*Friend.*—Why, what upon earth takes you there this time of year?

*Eb.*—To get my pension.

*Friend.*—Pension! why, for what?

*Eb.*—For services done the country.

*Friend.*—What! and how much? *Does it pay?*

*Eb.*—Yes, well. I get two cents for minding my own business, and two cents for letting other people's alone!

But I did not refer my fair inquisitor to Eb; and more pressing matters prevented a reply. I have thought since, that perhaps other readers may have had the same inquiry suggested.

